# Data-driven high-performance liquid chromatography method for the simultaneous analysis of disodium guanylate and disodium inosinate in mushrooms

**DOI:** 10.1016/j.dib.2025.111847

**Published:** 2025-08-05

**Authors:** Hilary Kwesi Ketemepi, Mohd Azrie Bin Awang, Wolyna Pindi, A. Sankara Narayanan, Nor Qhairul Izzreen M․N

**Affiliations:** a Faculty of Food Science and Nutrition, Universiti Malaysia Sabah, Jalan UMS, 88400 Kota Kinabalu, Sabah, Malaysia; b Food Security Research Laboratory, Faculty of Food Science and Nutrition, Universiti Malaysia Sabah, Jalan UMS, 88400 Kota Kinabalu, Sabah, Malaysia; c Department of Life Sciences, School of Science (SoS), GSFC University Fertilizer Nagar – 391750, Vadodara, Gujarat state, India; d Halal Services and Research Centre, Faculty of Food Science and Nutrition, Universiti Malaysia Sabah, Jalan UMS, 88400 Kota Kinabalu, Sabah, Malaysia

**Keywords:** Protein hydrolysate, *Hericium erinaceus*, Umami compounds, Flavour compounds, Bioactive peptides, Food industry, Food analysis

## Abstract

With the growing demand for high-throughput analyses that can detect diverse molecules with varying physicochemical properties in shorter times, researchers are increasingly focused on developing or modifying analytical methods. This is particularly relevant in the food, pharmaceutical/nutraceutical, cosmetic, agricultural, and environmental industries. This study aimed to modify, establish, and validate a high-performance liquid chromatography method with ultraviolet detection (HPLC-UV) for the simultaneous determination of disodium guanylate (GMP) and disodium inosinate (IMP) in mushrooms, using *Hericium erinaceus* as a model. These compounds are natural flavour enhancers that contribute to the umami taste, boost savoury flavour, and improve the palatability of mushroom-based dishes. Their presence in mushrooms also supports sodium reduction in recipes, making them valuable for healthier meal preparation. The optimal chromatographic conditions were determined using isocratic elution with a mobile phase composed of phosphate buffer, acetonitrile, and methanol in varying concentrations and pH levels. Flow rates and column temperatures were systematically tested. GMP and IMP were extracted from samples using deionized water, 0.1 M HCl, and 6 % acetic acid. Separation was carried out on a Kromasil 100–5-C18 column (4.6 × 250 mm; Sigma-Aldrich), with detection at 254 nm. The method showed excellent linearity (R² = 0.9989 for GMP and 0.9958 for IMP), low relative standard deviation (RSD: 1.07 % for GMP and 2.16 % for IMP), and good sensitivity, with LODs of 3.61 ppm (GMP) and 7.30 ppm (IMP) and LOQs of 10.93 ppm and 22.12 ppm, respectively. Recovery rates ranged from 91.4 % to 95.0 %, with RSDs below 5 %, indicating high accuracy and precision. Quantitative results revealed that *H. erinaceus* contains more IMP than GMP, contributing significantly to its umami profile. The validated method offers high precision, accuracy, and adaptability for other mushrooms and umami-rich foods. It is suitable for quality control, flavour enhancement, and nutritional profiling in the food industry—particularly in the development of natural flavouring agents. This method enables accurate quantification of umami-enhancing nucleotides, supporting flavour standardization, product formulation, and compliance with food labelling standards. It can also aid in shelf-life studies and thermal process optimization. Ultimately, this study advances natural flavour science by offering a robust method for analyzing GMP and IMP in *H. erinaceus.*

Specifications Table

**Table:** 

Subject	Food Science; Food Analysis
Specific subject area	Method development and Flavour analysis.
Type of data	Raw, Table, Graph, Figure, Analyzed, Processed, Chromatographs.
Data collection	Fresh *Hericium erinaceus* mushrooms were washed with distilled water, chopped into pieces, and dried in a hot air oven (ED056, BINDER, BINDER GmbH) until the moisture content reached approximately 7–10 % (wet basis). Details of the moisture determination process are provided in the method section. The dried mushrooms were ground into a fine powder using a laboratory grinding mill (LM 3610, Perten, PerkinElmer). The mushroom powder was sieved using a 0.35 mm stainless steel sieve (RETSCH 36,470,395), tightly packaged in aluminium bags, and stored at room temperature until further analysis. High-performance liquid chromatography with ultraviolet detection (HPLC-UV) (G1312B, 1200 Series, Agilent Technologies) was used for the analysis of disodium guanylate (GMP) and disodium inosinate (IMP). The separation was performed using a Kromasil 100–5-C18 column (4.6 × 250 mm; Sigma-Aldrich), with detection set at 254 nm, which was optimal for the target analytes. A systematic sample preparation procedure was employed. *H. erinaceus* powder was extracted using three different solvents: deionized water, 0.1 M HCl, and 6 % acetic acid (per gram of dry sample). After mixing and hydrolysis, the samples were cooled and centrifuged at 7500 rpm for 20 min at 20 °C. The supernatants were then filtered through a 0.45 µm Whatman polyethersulfone (PES) syringe filter to obtain clear filtrates for HPLC analysis.
	Calibration curves were constructed to evaluate linearity (R²), limit of detection (LOD), and limit of quantification (LOQ) for GMP and IMP. The relative standard deviation (RSD) of the slope of the standard curves was assessed to ensure data reliability. Although no specific inclusion or exclusion criteria were provided, the study focused exclusively on *H. erinaceus*, indicating an intention to investigate the nucleotide content of this mushroom species due to its culinary and nutritional relevance.
Data source location	*Hericium erinaceus* mushrooms of varying sizes were obtained from local markets/farmers in Kota Kinabalu, Sabah, Malaysia. They were tightly packaged in boxes and transported to the Faculty of Food Science and Nutrition-Universiti Malaysia Sabah.
Data accessibility	Repository name: Mendeley Data Data identification number: DOI: 10.17632/z295x2bb8y.1 Direct URL to data: https://data.mendeley.com/datasets/z295x2bb8y/1
Related research article	None

## 1 Value of the Data

• This study developed a modified HPLC-UV method for simultaneous determination of GMP and IMP in *H. erinaceus* mushrooms. Compared to conventional methods, such as HPLC with gradient elution or LC-MS/MS, the developed method offers a simpler setup, faster run time (under 15 min), and requires less expensive instrumentation.
• The method was validated with the reported LOD and LOQ values for GMP and IMP, making it suitable for detecting low-concentration nucleotides in complex food matrices. Researchers can replicate or adapt this method for food quality control, nutritional studies, and flavour enhancement across various mushroom species and food products.
• Calibration curves for GMP and IMP were included in this study to provide a transferable quantification framework applicable to future research. These methods can be adapted by researchers to develop standards for nucleotide analysis.
• Although this method uses water as the extraction solvent for simplicity and safety, its extraction efficiency may vary depending on the matrix composition, and should be optimized for different food types.
• This method supports the growing demand for accurate and rapid analysis of natural umami compounds. It has strong potential for flavour standardization, formulation of clean-label products, and development of natural flavouring agents.

## 2 Background

The presence of bioactive compounds in mushrooms has made them well-known as functional food ingredients that offer both nutritional benefits and sensory appeal to consumers [1]. Among these compounds, disodium guanylate (GMP) and disodium inosinate (IMP) are two important purine nucleotides that enhance umami flavour. Along with glutamate, these compounds contribute to making mushrooms a valuable plant-based source for flavour development [2,3]. However, the concentration of these umami-rich compounds varies depending on several factors, including mushroom species, cultivation conditions, maturity stage, anatomical parts, handling, processing methods, and storage time.

In addition to its well-documented health-promoting properties, *Hericium erinaceus* (commonly known as Lion’s Mane) has been identified by some researchers as a promising source of GMP and IMP. Nevertheless, accurately quantifying GMP and IMP in *H. erinaceus* remains challenging [4,5]. It is essential to determine the levels of these compounds in mushrooms to ensure the quality of mushroom-based functional foods and their contribution to umami flavour. Given the rising interest in natural, sustainable, and plant-based flavour enhancers, understanding the nucleotide composition of *H. erinaceus* could facilitate the development of mushroom-based umami seasonings and flavouring agents for the food industry.

## 3 Data Description

The dataset encompasses different components, as listed Tables in docx file, raw and processed data. xlsx, HPLC chromatograms for GMP standard solutions (PDF Files), and HPLC chromatograms for IMP standard solutions (PDF Files). They were grouped into five datasets (dataset 1–5).

Dataset 1 contain 5 tables. The summary of method development outcomes.pdf which is the first file (Table 1), contains a summary of method optimization for HPLC analysis of nucleotides (GMP and IMP). It includes details on optimized chromatographic conditions such as mobile phase composition: 10.0 mM KH₂PO₄ buffer : MeOH (90 % : 10 %, pH ≈ 4.60 ± 0.05), column: C18, 4.6 × 250 mm, flow rate: 0.8 mL/min, injection volume: 10.0 µL, detection wavelength: 254 nm, retention times: GMP ≈ 3.8 ± 0.1 min and IMP ≈ 4.0 ± 0.1 min, and performance metrics: resolution (R) = 2.1, asymmetry factor (AF) = 0.75–1.25, theoretical plates (N) > 10,000. The second file contains a table (Table 2) showing the extraction solvent type used, sample weight taken, volumes of the extraction solvents used, and concentration of the solvents. The results obtained during the flow rate optimization are summarized and outlined in Table 3, file 3. File 4 comprises the analytical validation parameters for GMP and IMP (Table 4). The data are organized into two tables that summarize the key performance characteristics of the method, including the accuracy, precision, LOD, and LOQ. Table 4a presents the accuracy and precision of the method evaluated through spike-recovery experiments at three concentration levels: low (10 ppm), medium (50 ppm), and high (90 ppm). Table 4b summarizes the sensitivity of the method, providing the slope (S) of the calibration curve and standard deviation (σ) of the response. From these values, the LOD and LOQ were calculated for both compounds. The performance of the HPLC methods in the current study was compared with that of existing protocols and is tabulated in Table 5, file 5.

Dataset 2 contains nine Excel files that contain raw and processed optimized parameters. These include flow rate optimization, injection volume optimization, mobile phase optimization, pH optimization, temperature optimization, summary of optimal analysis, summary of selected results, GMP std curve, LOD, LOQ, and RSD, and IMP std curve, LOD, LOQ, and RSD.

The HPLC chromatograms for GMP standard solutions (PDF Files) are contained in the third dataset and comprise a series of chromatograms for GMP standard solutions at different concentrations (5–100 ppm) obtained using the optimized HPLC method. Each file contained chromatographic conditions: mobile phase composition, flow rate, injection volume, and detection wavelength. The retention time and peak integration data for GMP at about 3.8 min. Also, the signal intensity and area percentage reports for quantification are provided. The files available in the dataset include GMP 5 ppm.pdf – Chromatogram and peak analysis for 5 ppm standard, GMP 10 ppm.pdf – Chromatogram and peak analysis for 10 ppm standard, GMP 20 ppm.pdf – Chromatogram and peak analysis for 20 ppm standard, GMP 30 ppm.pdf – Chromatogram and peak analysis for 30 ppm standard, GMP 40 ppm.pdf – Chromatogram and peak analysis for 40 ppm standard, GMP 50 ppm.pdf – Chromatogram and peak analysis for 50 ppm standard, GMP 60 ppm.pdf – Chromatogram and peak analysis for 60 ppm standard, GMP 70 ppm.pdf – Chromatogram and peak analysis for 70 ppm standard, GMP 80 ppm.pdf – Chromatogram and peak analysis for 80 ppm standard, GMP 90 ppm.pdf – Chromatogram and peak analysis for 90 ppm standard, and GMP 100 ppm.pdf – Chromatogram and peak analysis for 100 ppm standard.

The fourth dataset, which consists of HPLC chromatograms for IMP standard solutions (PDF Files), is similar to the third dataset. The main difference in the fourth dataset was the retention time and peak integration data for IMP at about 4.0 min.

The fifth and last dataset included three PDF files, each containing HPLC analysis results for samples labelled (a), (b), and (c) (PDF Files). These files provide detailed chromatographic data, including the peak retention times, areas, heights, and percentages. In addition, there is an Excel file for GMP and IMP conc.xlsx. The Excel file contains two sheets encompassing calculations of GMP and IMP concentrations based on peak areas from the HPLC data. A README file and the data were provided in Microsoft Word (.docx), Excel (.xlsx) and PDF formats and is accessible at https://data.mendeley.com/datasets/z295×2bb8y/1.

Data-driven optimization of our HPLC method significantly enhances both the accuracy and efficiency of GMP and IMP analyses in *H. erinaceus* compared to traditional techniques. By precisely defining retention times (GMP ≈ 3.8 ± 0.1 min; IMP ≈ 4.0 ± 0.1 min) and establishing robust calibration curves (r² > 0.99), we ensure high resolution and reproducibility of target peaks. This eliminates reliance on broader detection windows, which are commonly used in less-optimized protocols. Furthermore, the calculation of sensitivity parameters, including LOD and LOQ, enables trace-level quantification even in complex matrices, such as mushroom hydrolysates. Validation through spike recovery and inter-day precision analyses reinforced the reliability of the method. Collectively, these data-informed strategies provide a rigorous, reproducible, and scalable platform for nucleotide analysis in functional food applications.

## 4 Experimental Design, Materials and Methods

### 4.1 Sample preparation

Fresh fruiting bodies of *H. erinaceus* mushrooms of varying sizes were obtained from mushroom cultivators and suppliers in Malaysia. Upon receipt, the mushrooms were manually cleaned to remove soil and debris without washing, and then sliced into pieces (about 5 mm thick). Subsequently, the samples were dried in a hot air oven (ED056, BINDER, BINDER GmbH) until the moisture content was determined at about 7–10 % wet [6]. A laboratory grinding mill (LM 3610, Perten, PerkinElmer) was used to grind dried mushrooms into a fine powder. The mushroom powder was sieved using a 0.35 mm sieve (36,470,395, RETSCH, RETSCH GmbH), packaged tightly into aluminium bags, and stored at room temperature until further analysis.

### 4.2 Chemicals and reagents

All chemicals used were of analytical or HPLC grade. 5′-nucleotide standards consisting of disodium guanylate and disodium inosinate were procured from Sigma-Aldrich (USA). Potassium dihydrogen phosphate (KH_2_PO_4_), acetic acid (CH_3_COOH), hydrochloric acid (HCl) and sodium hydroxide (NaOH) were obtained from Merck (Germany). Organic solvents, including acetonitrile (ACN) and methanol (MeOH), were obtained from Fisher Scientific (USA). The chemicals were used to prepare the mobile phase and standard solutions for analysis. The 5′-nucleotide standards were dissolved in deionized water to create stock solutions, which were then diluted to appropriate concentrations for the experiments. All reagents and solvents were handled according to standard laboratory protocols to ensure accuracy and reproducibility.

### 4.3 Equipment

A high-performance liquid chromatography (HPLC) system (Agilent Technologies, 1200 Series, G1312B) equipped with a high-pressure binary pump SL was used. This pump allows precise control over solvent delivery and is capable of operating at pressures up to 400 bars (5800 psi). Due to its efficiency in separating nucleotides based on polarity and hydrophobicity, a Kromasil 100–5-C18 reverse-phase column (4.6 × 250 mm; Sigma-Aldrich) was used as the stationary phase. The analytes were separated at an optimized column temperature and detected using an ultraviolet (UV) detector set at 254 nm.

The system was configured to ensure optimal separation and detection of the analytes. The binary pump delivered solvents at a controlled flow rate, while maintaining the column temperature enhanced resolution. The UV detector provided accurate reading, allowing for the identification and quantification of the separated components.

### 4.4 Determination of moisture content

The moisture content of fresh *H. erinaceus* samples was determined using the air oven method based on the AOAC Official Method 925.10 (2005) with some modifications [6]. Before the analysis, the crucibles were washed and dried for about 30 min in a preheated oven to approximately 103 ± 5 °C. Afterwards, the crucibles were removed and allowed to cool in a desiccator. When the crucibles were at room temperature, they were removed, weighed, and recorded as the weight of crucible, *A*. Approximately 5 g of homogenized sample was weighed into the crucible and recorded as the weight of crucible + fresh sample, *B*. The crucibles and their contents were placed in an oven at about 103 ± 5 °C for 5 h. After drying, the crucibles were transferred to a desiccator and cooled. Upon reaching room temperature, the crucibles were weighed and recorded as the weight of the crucible + dried sample, *C*. All weights are presented in grams (g). Weight loss was reported as moisture using the following formula:

$$\%\text{Moisture}=\left(\frac{\left(\text{weight}\;\text{of}\;\text{crucible}+\text{fresh}\;\text{sample}\left(B\right)\right)-\left(\text{weight}\;\text{of}\;\text{crucible}+\text{dried}\;\text{sample}\left(C\right)\right)}{\left(\text{weight}\;\text{of}\;\text{crucible}+\text{fresh}\;\text{sample}\left(B\right)\right)-\left(\text{weight}\;\text{of}\;\text{crucible}\left(B\right)\right)}\times 100\right)$$

### 4.5 Production of *hericium erinaceus* protein hydrolysate

*H. erinaceus* mushroom powder was mixed with distilled water (substrate concentration (SC)) at a ratio of 1:20 (w/v), homogenized at room temperature to form a slurry and adjusted to pH 6.5. An enzyme substrate concentration (ESC) of 0.5 % (v/v) was prepared and the slurry mixtures were continuously heated in a water bath (Daihan MaXturdy 30) at 65 °C for 5 h. After hydrolysis, the mushroom slurry mixtures were transferred to a different water bath and heated at 85–95 °C for 10 min to inactivate enzyme activity. The hydrolyzed slurry mixtures were cooled to room temperature and centrifuged (EPPENDORF CENTRIFUGE 5430 R) at 7500 rpm at 20 °C for 20 min to remove any unwanted debris. Next, the supernatants of each mixture were carefully collected and freeze-dried using a FreeZone bulk tray dryer (18 L–50 °C, Labconco, Labconco Corporation) at −50 °C for 72 h to obtain *H. erinaceus* protein hydrolysate (HEPH) powder. The resulting hydrolysates were sealed with parafilm, labelled, and stored in closed glass bottles at 4–8 °C until further analysis. Mushroom protein hydrolysates (MPHs) for each treatment were produced in triplicate.

### 4.6 Extraction and determination of 5′-nucleotides

The method for assaying GMP and IMP in *H. erinaceus* was based on methods by Li et al. (2015), Zhang et al. (2022), Natalia and Rukmana (2018), and Poojary et al. (2017) with some modifications [[7], [8], [9], [10]]. Li et al. and Zhang et al. used deionized water as the extraction solvent, while Natalia and Rukmana used distilled water. Poojary et al. utilized 6 % acetic acid as the extraction solvent; however, they used 0.1 M HCl for experimental comparison.

The extraction protocol was optimized to maximize the recovery of GMP and IMP from *H. erinaceus* samples. Briefly, approximately 1 g of dried, homogenized mushroom powder was weighed into separate beakers and dissolved in different extraction solvents at varying volumes to produce different concentrations (Table 2). The samples were thoroughly mixed to ensure uniformity. The beakers were then placed in a water bath at 95 ± 5 °C for approximately 1 min to enhance nucleotide recovery from the mushroom matrix while preserving structural integrity [7]. After cooling, the mixtures were carefully transferred into centrifuge tubes and centrifuged at 7500 rpm at 20 °C for 20 min. The supernatants were then filtered through a Whatman 0.45 µm polyethersulfone (PES) syringe filter. The resulting clear filtrates were used directly for the detection and quantification of GMP and IMP. All sample preparations and analyses were conducted in triplicate.

Notably, while acid-based extractions can break down cellular matrices, they have been associated with partial degradation of labile purine nucleotides such as IMP and GMP under prolonged exposure. Moreover, extraction using dilute HCl is not suitable for food preparations and complicates downstream processing [10]. Based on trials with various solvents, deionized water was selected as the extraction solvent due to its safety, compatibility with biological systems, and effectiveness in extracting polar compounds such as nucleotides while minimizing structural degradation, as demonstrated by Li et al. (2015) and Zhang et al. (2022) [7,8]. Therefore, the adopted extraction conditions balanced efficiency, reproducibility, and compound stability for the targeted nucleotides in *H. erinaceus.*

The HPLC system consisted of a 10.0 µL sample loop, an ultraviolet (UV) detector, and a Kromasil 100–5-C18 column (4.6 × 250 mm, Nouryon). The column was maintained in a column oven at 30 °C. The mobile phase comprised 10.0 mM KH₂PO₄ and methanol (90 % : 10 %, v/v), adjusted to pH 4.60 ± 0.05, with a flow rate of 1.0 mL/min. Detection was carried out at 254 nm using a UV detector. Sample compounds were identified using standard 5′-nucleotides (disodium 5′-guanylate and disodium 5′-inosinate) based on their retention times. The analytes were quantified by comparing their peak areas with calibration curves constructed from standard compounds.

### 4.7 Standard preparation for nucleotide analysis

GMP and IMP standard stock solutions (1000 ppm) were individually prepared by dissolving appropriate amounts of each compound in deionized water. The stock solutions were stored at 4 °C. For calibration, working standards were freshly prepared daily by serial dilution of the stock solutions to obtain concentrations ranging from 5 to 100 ppm. Calibration curves were constructed by plotting the peak area against the concentration of each standard. Appropriate volumes of the selected mobile phase and 0.1 M HCl were used to mix the GMP and IMP standards, respectively.

The validity of the working standards and the method was demonstrated through the construction and analysis of standard calibration curves. Linearity (R²), limit of detection (LOD), and limit of quantification (LOQ) were evaluated from the calibration data. Additionally, the relative standard deviation (RSD) of the slope of the calibration curves was determined to assess reproducibility.

### 4.8 Mobile phase

The mobile phase was primarily composed of phosphate buffer due to the polar nature of the analytes to be assayed. The composition, concentration, and pH of the mobile phase were varied to monitor analytical parameters. The tested compositions included:

• 10.0 mM KH_2_PO_4_ (100 %) at pH 4.63
• 10.0 mM KH_2_PO_4_ : ACN (90 % :10 %) at pH 4.93
• 10.0 mM KH_2_PO_4_ : ACN (95 % : 5 %) at pH 4.57
• 10.0 mM KH_2_PO_4_ : MeOH (95 % : 5 %) at pH 4.76
• 10.0 mM KH_2_PO_4_ : MeOH (90 % : 10 %) at pH 4.60
• 10.0 mM KH_2_PO_4_ : MeOH (85 % : 15 %) at pH 4.58
• 20.0 mM KH_2_PO_4_ : MeOH (90 % : 10 %) at pH 4.65
• 0.5 mM KH_2_PO_4_ : MeOH (90 % : 10 %) at pH 4.48
• 5.0 mM KH_2_PO_4_ : MeOH (90 % : 10 %) at pH 4.55
• 15.0 mM KH_2_PO_4_ : MeOH (90 % : 10 %) at pH 4.70

The use of ACN resulted in closely eluting or overlapping peaks, which reduced resolution. In contrast, MeOH provided relatively better separation of the analytes (i.e., GMP and IMP, both possessing similar polarities). However, increasing the percentage of MeOH enhanced the elution strength of the mobile phase, leading to faster analyte elution. This decreased the interaction time of the analytes with the stationary phase, which likely caused peak overlap or poor resolution (i.e., reduced selectivity).

As a result, further optimization led to the selection of a mobile phase containing 10 % MeOH, which offered adequate resolution without excessive retention time when analyzing *H. erinaceus* samples. Additionally, KH₂PO₄ buffer maintained a stable pH, which is important for nucleotide solubility and stability. This buffer also provided consistent ionic strength, ensuring the reproducibility and accuracy of the developed method.

Furthermore, phosphate buffers are compatible with various detection methods, as they do not interfere with UV spectrophotometric readings and have minimal absorbance at 254 nm—commonly used for nucleotide detection [11]. In contrast to the sodium hexanesulfonate buffer system used by Natalia and Rukmana (2018), the current study selected potassium phosphate buffer with methanol to avoid column fouling and the poor reproducibility often associated with ion-pairing agents. The chosen system achieved adequate retention and resolution of the target nucleotides while maintaining method simplicity and compatibility with standard HPLC protocols.

Prior to use, the mobile phase was filtered through a 0.22 µm membrane and degassed via sonication. Flow rates of 0.5, 0.8, and 1.0 mL/min were tested, and analyte elution was monitored at 20, 30, and 40 °C, with UV detection set at 254 nm. The pH of the mobile phase solutions ranged from 4.48 to 4.92. Additionally, two mobile phases of 10.0 mM KH₂PO₄ : MeOH (90 % : 10 %) were prepared with pH adjustments to acidic (pH 2.53) and neutral (pH 7.51) conditions. Peak sharpness, retention times, and resolution of GMP and IMP were evaluated to verify the method’s optimization.

### 4.9 Optimization strategies

The HPLC method was developed using a systematic, data-driven approach to optimize key parameters for the simultaneous detection of GMP and IMP. Several mobile phase compositions were evaluated, including 10.0 mM KH₂PO₄ buffer mixed with methanol at varying ratios (95 % : 5 %, 90 % : 10 %, and 85 % : 15 %, v/v). In addition, pH values ranging from 2.53 to 7.51 were tested. A composition of 10.0 mM KH₂PO₄ : MeOH (90 % :10 %, v/v) at a pH of approximately 4.60 ± 0.05 provided the best resolution, peak shape, and reproducibility, and was therefore selected for further analysis.

The flow rate was optimized by testing values ranging from 0.5 to 1.0 mL/min (Table 3). A flow rate of 0.8 mL/min offered optimal chromatographic performance, characterized by minimal peak tailing, acceptable backpressure, and a short analysis time (<5 min). Column temperature was also optimized by testing at 20, 30, and 40 °C. A temperature of 20 °C resulted in longer retention times, while 30 and 40 °C moderately reduced the retention times. A column temperature of 30 °C was selected as it provided the best balance between peak resolution, retention time stability, and baseline separation. This is consistent with the conditions reported by Li et al. and Zhang et al. in their studies [7,8]. Under the optimized conditions, the final retention times were approximately 3.8 ± 0.1 min for GMP and 4.0 ± 0.1 min for IMP, with a resolution of 2.1.

The modified HPLC method offers several improvements over existing methodologies for nucleotide analysis in mushrooms. Notably, GMP and IMP can be rapidly and efficiently separated with retention times of 3.8 ± 0.1 min and 4.0 ± 0.1 min, respectively. Additionally, the total analysis time is significantly shorter—<10 min—compared to conventional methods that often exceed this duration [12]. A C18 reversed-phase column (4.6 × 250 mm) was used with an isocratic mobile phase consisting of 10.0 mM KH₂PO₄ buffer and methanol (90 % : 10 %, v/v) at pH 4.60 ± 0.05, ensuring a stable baseline. This eliminated the need for gradient elution or complex solvent systems, thereby reducing method complexity and cost.

**Fig. 1:**
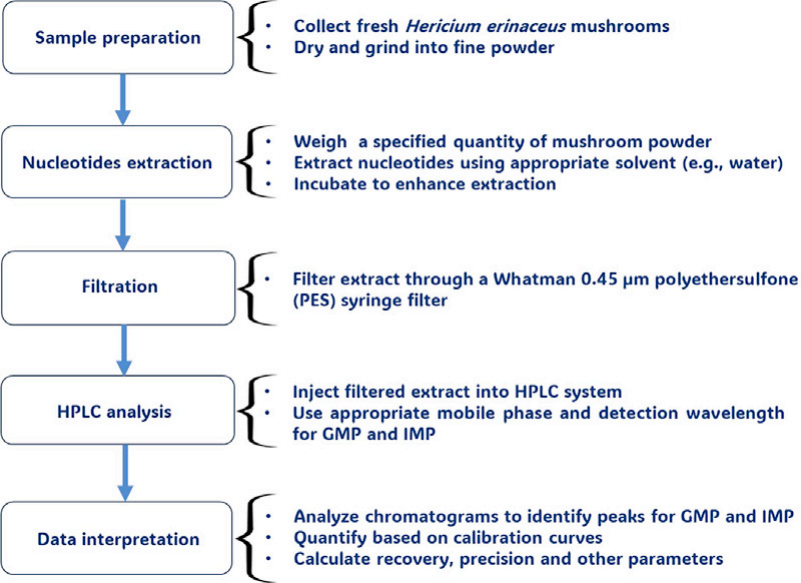
Flowchart of sample preparation and nucleotides analysis using HPLC-UV.

In terms of sensitivity, the method achieved low limits of detection (LODs) for GMP (3.61 ppm) and IMP (7.30 ppm), with corresponding limits of quantification (LOQs) of 10.93 ppm and 22.12 ppm, respectively. This makes the method suitable for trace-level quantification of nucleotides in mushroom matrices. Due to its versatility, the method can be applied to emerging functional food studies, such as those involving *H. erinaceus*, a mushroom species less commonly targeted for nucleotide profiling. The workflow of sample preparation and nucleotide analysis using HPLC is illustrated in Fig. 1.

### 4.10 Method validation

#### 4.10.1 Linearity, accuracy, precision, LOD and LOQ

The developed HPLC method was validated for the quantitative determination of GMP and IMP. Calibration curves were established by plotting the peak area (mAU*s) against analyte concentration (ppm). GMP exhibited a linear regression of *y* = 14.668x – 0.7351 with an R² value of 0.999, while IMP showed a regression of *y* = 13.737x – 32.249 and R² = 0.9958 (Dataset 2), confirming strong linearity in the range of 5–100 ppm.

Accuracy was evaluated through spike recovery at low (10 ppm), medium (50 ppm), and high (90 ppm) levels, analyzed in triplicate. Recovery rates ranged from 91.4 % to 95.0 % for IMP and from 92.8 % to 94.6 % for GMP, indicating satisfactory analyte recovery from the sample matrix.

Precision was determined by inter-day repeatability across three days. The relative standard deviation (RSD) values ranged from 4.57 % to 4.75 % for all concentrations, demonstrating acceptable inter-day variability and consistent performance of the analytical method which supports the robustness and reproducibility of the method (Table 4a).

The limits of detection (LOD) and quantification (LOQ) were calculated based on the standard deviation of the response and the slope of the calibration curve (LOD = 3.3σ/S; LOQ = 10σ/S). The estimated LODs were 3.61 ppm for GMP and 7.30 ppm for IMP, while LOQs were 10.93 ppm and 22.12 ppm, respectively, indicating the method’s suitability for detecting trace levels of these analytes (Table 4b).

### 4.11 Method comparison with recognized protocols

To further establish the analytical reliability of the proposed HPLC method for GMP and IMP determination in *H. erinaceus*, its performance was compared with that reported in peer-reviewed studies. Comparative metrics included recovery rates, relative standard deviation (RSD), limits of detection (LOD), limits of quantification (LOQ), and chromatographic conditions.

The proposed method exhibited excellent recovery for both GMP (92.8–94.6 %) and IMP (91.4–95.0 %), with RSD values below 5 %. The calculated LODs and LOQs were 3.61 ppm and 10.93 ppm for GMP, and 7.30 ppm and 22.12 ppm for IMP, respectively. When compared with established methods, such as those described by Poojary et al. (2017), Zhang et al. (2022), and Li et al. (2015), the present method demonstrated comparable sensitivity and precision while employing a simpler UV detection protocol. A summary of the comparative analysis is presented in Table 5.

### 4.12 Simultaneous separation and quantification in mushroom matrices

The modified method ensures the simultaneous separation and quantification of GMP and IMP with minimal interference through the following strategies:

• *Choice of stationary phase*: The use of a C18 reversed-phase column (4.6 × 250 mm) provided sufficient hydrophobic interaction to retain GMP and IMP while allowing good resolution from matrix components.
• *Mobile phase composition and* pH: The selected mobile phase of 10.0 mM KH₂PO₄ : MeOH (90 % : 10 %, v/v, pH ≈ 4.60 ± 0.05) enabled optimal ionic interaction and polarity balance, allowing sharp, separated peaks even in the presence of other mushroom-derived compounds.
• *Sample clarification*: Mushroom samples were carefully prepared via aqueous extraction, followed by filtration through a 0.22 µm microporous membrane to remove particulate matter and reduce background noise.
• *Selectivity confirmation*: Chromatographic runs of blank mushroom extracts (without GMP/IMP standards) were performed to ensure that there were no overlapping or interfering peaks near GMP and IMP retention times. No significant interference was observed at 3.8 and 4.0 min respectively.

This confirms the method’s selectivity, making it suitable for routine application in complex mushroom matrices, such as *H. erinaceus* hydrolysates.

### 4.13 Troubleshooting tips

The common issues encountered during nucleotide extraction and HPLC analysis are summarized below:

• Low peak resolution: Ensure that the mobile phase is freshly prepared and degassed properly. Check column integrity.
• Sample precipitation: Filter the samples through a Whatman 0.45 µm polyethersulfone (PES) syringe filter before injection.
• Mobile phase: Filter the mobile phase through a 0.22 µm membrane filter and degassed by sonication before use.
• Peak retention shifts: Monitor pH of the mobile phase and re-adjust if necessary.
• Baseline noise or drift: Use HPLC-grade solvents and verify UV lamp condition.

## Limitations

This study focuses on the analysis of GMP and IMP in *H. erinaceus* mushrooms. Although its applicability to other mushroom species or food matrices has not yet been established, the method can be adapted with appropriate modifications where necessary.

While machine learning (ML) was not employed in the current method development, future integration of ML-driven optimization could enhance efficiency. As analytical workflows become increasingly automated, issues such as data security and energy consumption—highlighted in studies like “Systematic Poisoning Attacks on and Defenses for Machine Learning in Healthcare [13]” and “Energy-Efficient Long-term Continuous Personal Health Monitoring [14]”—will become increasingly important. Although these aspects are beyond the scope of this study, they merit further exploration.

For industrial-scale applications, throughput can be improved using ultra-high-performance liquid chromatography (UHPLC) systems with sub-2 µm particle columns. Automation of sample handling and injection may further enhance reproducibility and reduce manual workload [15]. Implementing solvent recycling systems or using greener mobile phases would help reduce operational and environmental impacts. Inline chromatographic detection could also enable real-time monitoring of mushroom extracts or seasoning production. Finally, validating the method under recognized industry standards such as ISO 17,025 or FDA Good Laboratory Practice (GLP) would support its broader application in both laboratory and production settings.

## Ethics Statement

This work does not involve human subjects and animal experiments.

## CRediT Author Statement

**Hilary Kwesi Ketemepi:** Conceptualization, methodology, data curation, investigation, formal analysis, writing – original draft, writing – review and editing, **Mohd Azrie Awang:** Supervision, writing – review and editing, **Wolyna Pindi:** Writing – review and editing, **A. Sankara Narayanan:** Writing – review and editing, **Nor Qhairul Izzreen Mohd Noor:** Conceptualization, methodology, supervision, project administration, writing – review and editing, validation.

## Data Availability

Mendeley Datamethod modification (Original data).

## References

[1] Ketemepi H.K., Bin Awang M.A., Seelan J.S.S., Noor N.Q.I.M. (2024). Extraction process and applications of mushroom-derived protein hydrolysate: a comprehensive review. Futur. Foods.

[2] Xia R. (2023). Energy status regulated umami compound metabolism in harvested shiitake mushrooms (Lentinus edodes) with spores triggered to release. Food Sci. Hum. Wellness.

[3] Zhao J., Wang T., Zhang C., Han X., Yan J., Gan B. (2023). A comparative analysis of the umami taste of five fresh edible mushrooms by simulating the chemical environment of oral digestion in vitro. LWT.

[4] Tripodi F. (2022). Anti-aging and neuroprotective properties of Grifola frondosa and Hericium erinaceus extracts. Nutrients.

[5] Szućko-Kociuba I., Trzeciak-Ryczek A., Kupnicka P., Chlubek D. (2023). Neurotrophic and neuroprotective effects of hericium erinaceus. Int. J. Mol. Sci..

[6] C. A. AOAC (2005). Official methods of analysis of the association of analytical chemists international. Off. Methods Gaithersbg. MD USA.

[7] Li X. (2015). Effects of drying methods on the tasty compounds of Pleurotus eryngii. Food Chem..

[8] Zhang W. (2022). Characterization of Pleurotus citrinopileatus hydrolysates obtained from Actinomucor elegans proteases compared with that by commercial proteases. J. Food Sci..

[9] Natalia D.K., Rukmana T.I. (2018). Development of a high-performance liquid chromatography method for analyzing disodium 5’-guanylate and disodium 5’-inosinate levels in flavor enhancers. Int. J. Appl. Pharm..

[10] Poojary M.M., Orlien V., Passamonti P., Olsen K. (2017). Improved extraction methods for simultaneous recovery of umami compounds from six different mushrooms. J. Food Compos. Anal..

[11] Mauger J.W. (2017). Physicochemical properties of buffers used in simulated biological fluids with potential application for in vitro dissolution testing: a mini-review. Dissolut. Technol..

[12] Sommer I., Schwartz H., Solar S., Sontag G. (2010). Effect of gamma-irradiation on flavour 5′-nucleotides, tyrosine, and phenylalanine in mushrooms (Agaricus bisporus). Food Chem..

[13] Mozaffari-Kermani M., Sur-Kolay S., Raghunathan A., Jha N.K. (2014). Systematic poisoning attacks on and defenses for machine learning in healthcare. IEEE J. Biomed. Heal. Inform..

[14] Nia A.M., Mozaffari-Kermani M., Sur-Kolay S., Raghunathan A., Jha N.K. (2015). Energy-efficient long-term continuous personal health monitoring. IEEE Trans. Multi-Scale Comput. Syst..

[15] Kaplitz A.S. (2019). High-throughput and ultrafast liquid chromatography. Anal. Chem..

